# Behavioural response of mosquito vectors *Aedes aegypti*, *Anopheles stephensi* and *Culex quinquefasciatus* to synthetic pyrethroid and organophosphorus-based slow-release insecticidal paint

**DOI:** 10.1186/s13071-021-04746-x

**Published:** 2021-05-17

**Authors:** Sunil Dhiman, Kavita Yadav, B. N. Acharya, Raj Kumar Ahirwar, D. Sukumaran

**Affiliations:** Defence Research and Development Establishment, Jhansi Road, Gwalior, Madhya Pradesh 474002 India

**Keywords:** SRIP, Mosquitoes, Insecticide, Excito-repellency, Mortality

## Abstract

**Background:**

The direct toxicological impact of insecticides on vector mosquitoes has been well emphasized; however, behavioural responses such as excito-repellency and physical avoidance as a result of insecticide exposure have not been much studied. We have demonstrated the excito-repellency and behavioural avoidance in certain vector mosquito species on exposure to a slow-release insecticidal paint (SRIP) formulation in addition to direct toxicity.

**Methods:**

A SRIP formulation developed by the Defence Research and Development Establishment, Gwalior, contains chlorpyriphos, deltamethrin and pyriproxyfen as active insecticides. *Anopheles stephensi, Culex quinquefasciatus* and *Aedes aegypti* mosquitoes were used to study the excito-repellency response of the formulation. The experiments were performed in a specially designed dual-choice exposure and escape chamber made of transparent polymethyl methacrylate. For the experiments, the SRIP formulation was applied undiluted at a rate of 8 m^2^ per kg on 15 cm^2^ metallic surfaces. Mosquitoes were introduced into the exposure chamber, and observations of the movement of mosquitoes into the escape chamber through the exit portal were taken at 1-min intervals for up to 30 min.

**Results:**

The evaluated formulation displayed strong excito-repellency against all three tested vector mosquito species. Results showed that the ET_50_ (escape time 50%) for *Ae. aegypti*, *An. stephensi* and *Cx. quinquefasciatus* was 20.9 min, 14.5 min and 17.9 min for contact exposure (CE) respectively. Altogether in CE, the escape rates were stronger in *An. stephensi* mosquitoes at different time intervals compared to *Ae. aegypti* and *Cx. quinquefasciatus* mosquitoes. The probit analysis revealed that the determined ET did not deviate from linearity for both non-contact exposure (NCE) and placebo exposure (PE) (χ^2^ ≤ 7.9; *p* = 1.0) for *Ae. aegypti* mosquitoes and for NCE (χ^2^ = 8.3; *p* = 1.0) and PE (χ^2^ = 1.7; *p* = 1.0) treatments in *Cx. quinquefasciatus*. Mortality (24 h) was found to be statistically higher (F = 6.4; *p* = 0.02) in *An. stephensi* for CE but did not vary for NCE (*p* ≥ 0.3) and PE (*p* = 0.6) treatments among the tested mosquito species. Survival probability response suggested that all the three tested species displayed similar survival responses for similar exposures (χ^2^ ≤ 2.3; *p* ≥ 0.1).

**Conclusion:**

The study demonstrates the toxicity and strong behavioural avoidance in known vector mosquito species on exposure to an insecticide-based paint formulation. The combination of insecticides in the present formulation will broaden the overall impact spectrum for protecting users from mosquito bites. The efficacy data generated in the study provide crucial information on the effectiveness of the tested formulation and could be useful in reducing the transmission intensity and disease risk in endemic countries.
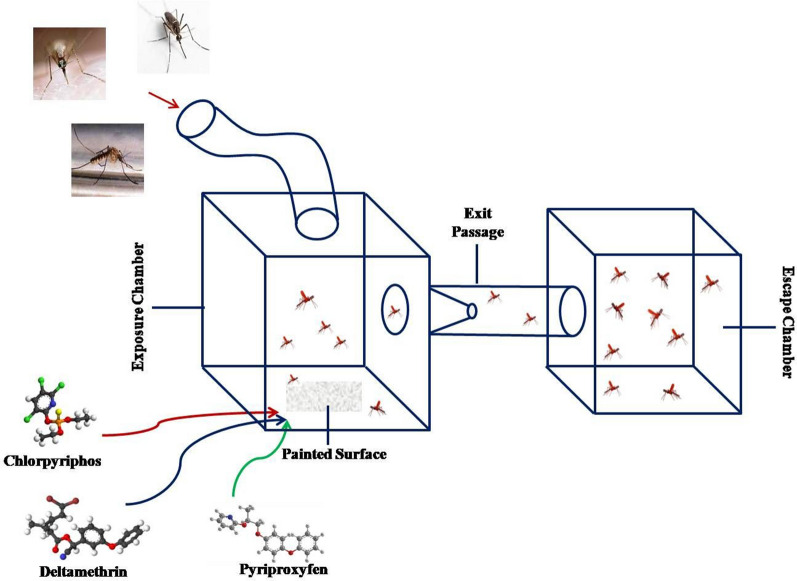

## Background

Mosquito-borne diseases affect millions of people globally [1]. Mosquitoes belonging to *Anopheles*, *Aedes* and *Culex* genera are considered important because of their involvement in transmitting many deadly diseases among humans. *Anopheles* caused 229 million confirmed malaria cases and 0.41 million deaths during 2019 in about 87 countries [1]. Day-biting *Aedes* mosquitoes are now distributed over 50% of world's inhabited area and uninterruptedly support the transmission of many viral diseases [2, 3]. *Culex* mosquitoes are principally vectors of bancroftian filariasis, but also capable of spreading flaviviruses [4, 5] as well as alphaviruses [6]. Tropical and sub-tropical countries are at continuous risk of different vector-borne diseases including malaria, dengue, filariasis, etc., and report considerable morbidity and mortality associated with these infections annually. Despite extensive research on mosquito control for decades, the prevention of these vectors primarily relies on using insecticides as residual sprays, space sprays and larvicides.

Although existing mosquito control strategies have been useful in reducing the disease incidence in many endemic countries, newer tools and methods are needed to effectively enhance the insecticide exposure to mosquitoes while protecting humans. The objective of intervention programmes is to minimise the contact between the mosquito and human host and at the same time to contain the vector population. Intervention strategies based on repelling vector mosquitoes from human-inhabited areas remain the area of investigation as they have contributed recognisably to reducing mosquito-borne disease incidence in different endemic settings [7].

Among the insecticides used in various vector interventions methods, such as indoor residual spray and long-lasting insecticidal nets (LLINs), synthetic pyrethroid-based formulations remain the key components and are widely accepted because of the high toxicity to mosquitoes and very low toxicity to higher animals including humans. In addition to their use in bed nets, pyrethroids are also gaining popularity for impregnation of fabric materials for use as wearable clothes and insect-repelling curtains, etc. [2]. Synthetic pyrethroids exert insecticidal effects by acting on the voltage-gated sodium channel (VGSC) present on the neural membrane of mosquitoes. However, their low concentration in the atmosphere acts as a spatial repellent against different mosquito species [8]. Additionally, pyrethroids cause contact irritation to the mosquitoes through excito-repellency, which is a more important and effective phenomenon when pyrethroids are present on surfaces [7, 8]. Pyrethroids used on surfaces result in excitation and hence force pre-mature and quick movement of insects from the treated areas. Some studies have suggested that this mechanism of repellency exploiting mosquito behaviour is still more effective in the resistant populations of vector mosquitoes [9–12].

Available literature suggests that most studies have focused on the direct toxicological effect of insecticides such as knockdown and mortality on mosquito populations. However, behavioural responses of mosquito vectors to insecticide formulations exposure have not been much explored and emphasized [13, 14]. Some of the previous studies have shown that insecticides at different concentrations exert an excito-repellency effect on mosquitoes and other arthropods [9, 15–18]. An earlier study has demonstrated that females of the malaria vector *An. minimus* rapidly escaped from exposure chambers after direct contact with the insecticides DDT, deltamethrin and lambda-cyhalothrin [15]. The study further showed that the non-contact repellency response to the insecticides was appreciable, but not equally pronounced in different test species [15]. Sukkanon et al. [19] showed that mosquito vectors *Ae. aegypti*, *An. minimus* and *An. dirus* displayed considerable excito-repellency responses in both contact and non-contact exposure to transfluthrin. The study further showed that a transfluthrin-resistant population of *Ae. aegypti* also displayed significant escape on exposure [19]. Delayed mortality (> 95%) has been reported in mosquitoes that were caged at a sufficient distance from the pyrethroid-treated surface and not allowed to contact the insecticide [17, 18].

The insecticide formulations exhibiting excito-repellency could provide better and prolonged protection from mosquito bites and are also thought to delay resistance development in mosquitoes due to reduced or no contact with the insecticide [20]. Therefore, the insecticidal formulations having an excito-repellency effect on different arthropod vectors in addition to normal insecticidal activity could be useful in vector control programmes. In the present study synthetic pyrethroid and organophosphate-based slow-release insecticidal paint was assessed for excito-repellent activity against mosquito vectors *Aedes aegypti*, *Anopheles stephensi* and *Culex quinquefasciatus*.

## Method

### Insecticidal paint

Slow-release insecticidal paint (SRIP) is an improved paint formulation developed by the Defence Research and Development Establishment (DRDE), Gwalior, India. It contains synthetic pyrethroid (SP) deltamethrin (1%), the organophosphate (OP) chlorpyriphos (0.5%) and the pyridine-based insect growth regulator (IGR) pyriproxyfen (0.075%) as active ingredients. The formulation is alkyd resin based, where the active ingredients (SP, OP and IGR) are embedded throughout the continuous solvent phase. The insecticide residing in the resin matrix is released gradually to the outer surface, providing long-term efficacy against mosquitoes and other insects. The toxicity studies of this formulation conducted at a GLP-accredited laboratory suggest that the formulation is safe to mammals. The formulation has been made flame retardant for the specific use of the Indian armed forces. All the active ingredients used in the formulations have been approved by WHO for use in vector control programmes and have been used in different vector control formulations [16, 17, 21].

### Test mosquitoes

For the present study, *An. stephensi* and *Cx. quinquefasciatus* mosquitoes were taken from the laboratory insectary of the Vector Management Division at Defence Research and Development Establishment (DRDE), Gwalior (India). Insecticide-sensitive strains of mosquito species are maintained in the laboratory insectary at the recommended temperature (27 ± 2 °C) and relative humidity (75–80%) and reared in standard wooden cages. The mosquito larvae were fed on yeast powder, whereas adults were provided with sucrose solution (10%) in soaked cotton *ad libitum. Ae. aegypti* mosquitoes were colonized from wild-caught larvae collected from the field [22] in the Gwalior area (26°13′5.8332″N and 78°10′58.1916″E). Generations of the colonised *Ae. aegypti* were reared and used for the present study.

### Behaviour response assays

**Fig. 1:**
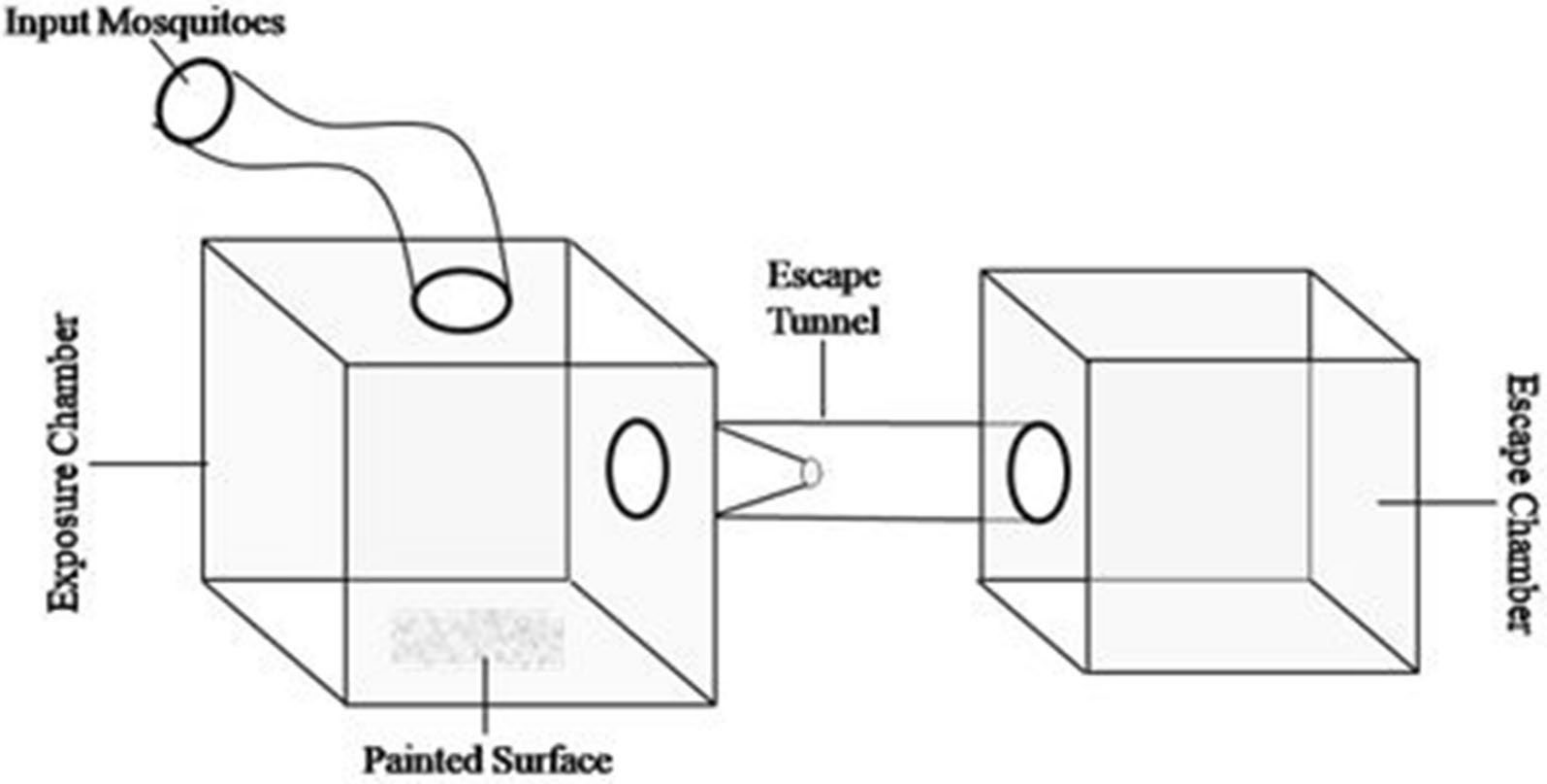
Experimental set up for assessing the excito-repellency effect of the slow-release insecticidal paint formulation

Excito-repellency response assays were performed using 5–7-day-old females, used in cohorts of approximately 20–30 mosquitoes. Test mosquitoes were segregated about 24 h before the actual experiments and were only provided with water-soaked cotton (sugar starved). Experiments were conducted in custom-designed dual-choice excito-repellency chambers [23, 24]. The complete experiment setup (Fig. 1) was made up of transparent polymethyl methacrylate (PMMA) and essentially comprised an exposure chamber (30 × 30 × 30 cm), an escape chamber (20 × 20 × 20 cm) and an escape tunnel (10 cm diameter; 30 cm length) connecting both chambers. Test material (SRIP formulation) was applied undiluted at a rate of 8 m^2^/kg on 15 cm^2^ metallic surfaces using a regular paint brush and dried at room temperature for 48 h before actual exposures. The treated surface was placed on the floor of the exposure chamber to observe the mosquito movement towards the escape trap in response to the chemical stimulus or contact irritancy due to insecticidal paint. Cohorts of test mosquitoes were introduced into the exposure chamber using a glass aspirator (WHO Centre, Universiti Sains Malaysia) and left for 30 s, and observations on the movement of mosquitoes into the escape chamber through the exit portal were taken at 1-min intervals for up to 30 min. Mosquitoes that did not show any movement were also recorded for each minute interval. All the living and presumed dead mosquitoes were maintained individually in paper cups (100 ml capacity) to score the mortality after 24 h of experiments. In this study, contact exposure (CE) (where mosquitoes have the choice to come in contact with the treated surface), non-contact exposure (NCE) (where the treated surface was covered with wire mesh) and placebo exposure (PE) treatment designs were used to determine the excito-repellency response of the vector mosquitoes.

### Quantification of active ingredients

Approximately 200 mg of the paint was scraped from the metallic surfaces, weighed and transferred to a 20-ml volumetric flask. To the volumetric flask, 10 ml of acetonitrile (HPLC grade, Merck) was added and sonicated for 10 min. The volume was made up to 20 ml by adding acetonitrile and the flask was shaken gently to attain uniformity. One millilitre of the obtained mixture was drawn and filtered through a 0.22-μ PVDF membrane filter and stored at − 4 °C for HPLC studies.

Active compounds were separated on a C18 reversed-phase column (4.6 × 250 mm, particle size 5 μm; Waters XTerra™) maintained at 25 °C. Methanol and water in the ratio of 80:20 (v/v) were chosen as the mobile phase at a flow rate of 1 ml/min using a Waters 1225 binary pump. Detection was carried out by a Waters 2487 dual-absorbance detector tuned at 280 nm. Samples were injected through a Rheodyne injector, and the injection volume was 20 μl. The instrument used the Empower software (Europa Science, Ltd., Cambridge, UK) for data collection and overall instrument control.

### Statistical analysis

Comparisons among the escaped mosquitoes for contact, non-contact and placebo treatments at different time intervals were performed using ANOVA followed by Tukey test of multiple comparisons. Difference in the escape behaviour between different exposures was determined using Student’s *t*-test. A probit model has been used to determine the escape time (ET) using the count of mosquitoes that moved into the escape chamber. Survival analysis using the Kaplan-Meier method was performed to analyse the behavioural response of the tested mosquito species. Non-overlapping 95% confidence intervals were used to determine the significant differences among the treatments (*p* < 0.05).

## Results

### Escape response and probit analysis

**Fig. 2:**
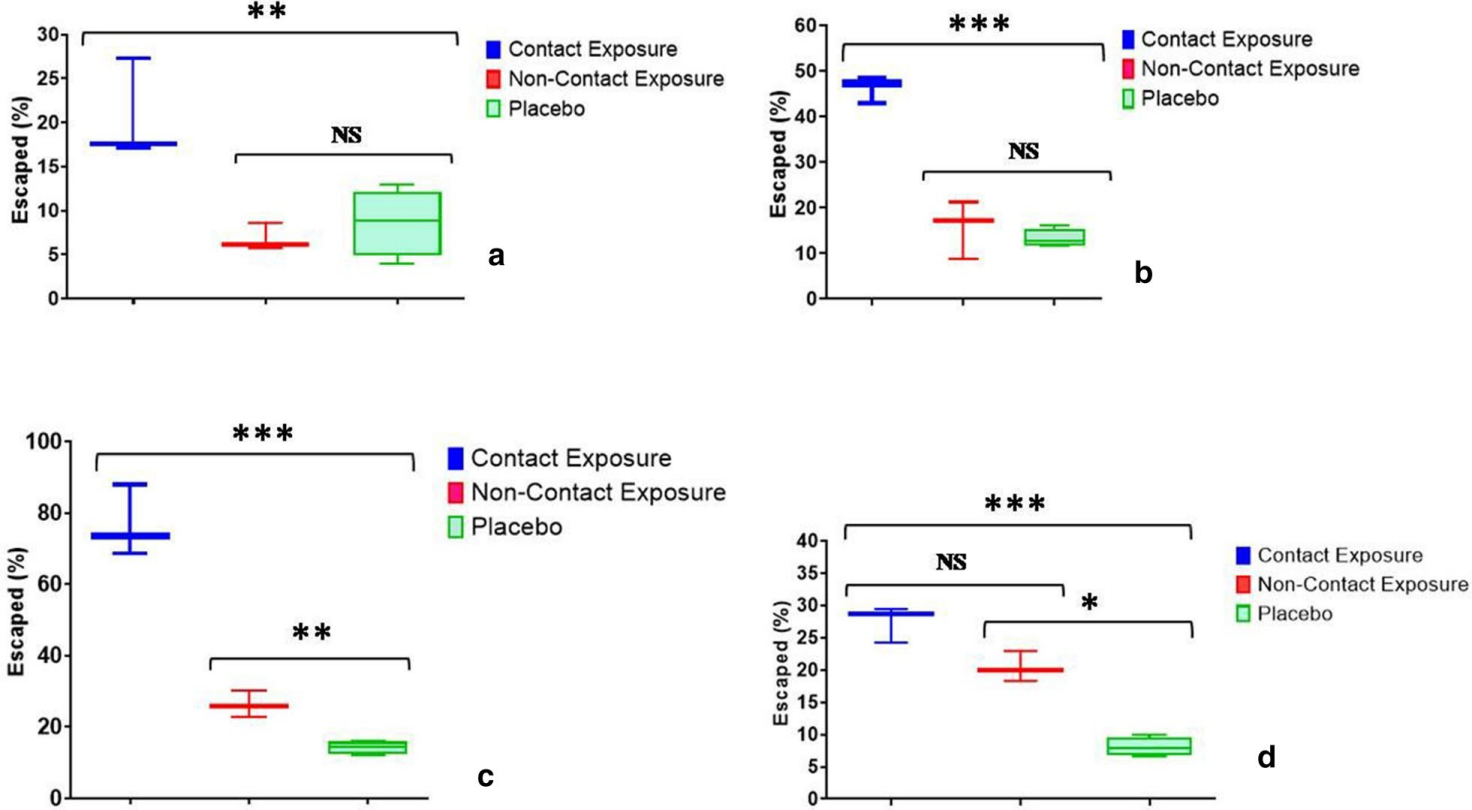
Box-whisker plot displaying the escape % of *Aedes aegypti* mosquitoes **a** 10 min post-exposure, **b** 20 min post-exposure, **c** 30 min pos- exposure; **d** 24-h mortality (*p < 0.05; **p < 0.001; ***p < 0.0001; *NS* non-significant)

In dengue vector *Ae. aegypti*, after 10 min of exposure, the escape % in CE was 20.7 ± 3.3 and was found to be higher compared to NCE and PE (F = 10.8; *p* = 0.007; R^2^ = 0.76) (Table 1). Similar results were recorded after 20 min and 30 min of exposure as escape % in CE was 46.2 ± 1.7 (F = 66.9; *p* < 0.0001; R^2^ = 0.95) and 76.7 ± 5.8 (F = 104.2; *p* < 0.0001; R^2^ = 0.97) respectively (Table 1; Fig. 2). However, it was observed that the escape % was similar in NCE and PE after 10 min and 20 min of exposure (t < 0.63; *p* > 0.59), whereas it was found to be higher in NCE (t = 13.1; *p* = 0.006) after 30 min of exposure (Table 1). The results showed that ET_50_ for CE, NCE and PE was 20.9 min (95% CI: 18.9–23.7), 95.8 min and 261.1 min respectively (Table 2). The probit analysis revealed that ET determined for *Ae. aegypti* mosquitoes did not deviate from linearity for both NCE and PE (χ^2^ ≤ 7.9; *p* = 1.0).

**Table 1:** Escape and mortality in test mosquito species in contact, non-contact and placebo exposure of slow-release insecticidal paint (SRIP) formulation

Exposure	Mosquito species (N)	Escape % 10 min (mean ± SEM)	95% CI	Escape % 30 min (mean ± SEM)	95% CI	M_24_ h	95% CI
Contact (CE)	*Ae. aegypti* (102)	20.7 ± 3.3	6.4–34.9	76.7 ± 5.8	51.8–101.6	27.4 ± 1.6	20.4–34.4
	*An. stephensi* (125)	35.2 ± 4.7	20.3–50.1	82.0 ± 5.9	63.0–100.9	39.2 ± 4.9	23.6–54.7
	*Cx. quinquefasciatus* (110)	25.8 ± 2.6	17.5–34.2	77.2 ± 1.9	71.2–83.2	23.5 ± 1.4	18.9–28.1
Non-contact (NCE)	*Ae. aegypti* (103)	6.8 ± 0.9	2.9–10.7	26.3 ± 2.2	17.0–35.6	20.4 ± 1.4	14.5–26.3
	*An. stephensi* (127)	9.0 ± 2.1	2.3–15.8	39.6 ± 4.4	25.6–53.7	25.1 ± 4.3	11.5–38.6
	*Cx. quinquefasciatus* (125)	14.2 ± 1.3	10.2–18.2	37.9 ± 3.7	26.1–49.7	18.8 ± 2.1	12.0–25.6
Placebo (PE)	*Ae. aegypti* (112)	8.7 ± 1.9	2.7–14.6	14.2 ± 0.9	11.2–17.2	8.1 ± 0.7	5.7–10.4
	*An. stephensi* (127)	10.6 ± 2.9	1.5–19.7	19.5 ± 1.3	15.3–23.7	9.7 ± 1.6	4.7–14.7
	*Cx. quinquefasciatus* (114)	9.1 ± 2.9	0.4–18.9	21.7 ± 1.9	15.6–27.8	9.0 ± 1.1	5.6–12.4

SEM, standard error of mean; Escape % 10 min/30 min, mosquitoes escaped in 10/30 min; CI, confidence interval; M_24_, mortality after 24 h; N, total number; CE/NCE/PE, contact/non-contact/placebo exposure

**Table 2:** Excito-repellency response of *Ae. aegypti*, *An. stephensi* and *Cx. quinquefasciatus* against the slow-release insecticidal paint (SRIP) formulation

Species	ET_10_ (95%CI)	ET_50_ (95% CI)	ET_99_ (95% CI)	Slope (±)	χ^2^ (*p*)	h	g	r
*Ae. aegypti* (CE)	4.8 (3.9–5.5)	20.9 (18.9–23.7)	298.9 (238.9–461.4)	2.0 ± 0.1	93.4 (0.0)	3.3	0.3	0.9
*Ae. aegypti* (NCE)	11.6 (10.2–12.9)	95.8 (72.7–141.2)	4414.0 (1907.0–14,593.0)	1.4 ± 0.1	5.1 (1.0)	0.2	0.0	1.0
*Ae. aegypti* (PE)	14.9 (12.9–17.0)	261.1 (151.5–625.1)	47,361.2 (10,263.3–561,000.7)	1.0 ± 0.1	7.9 (1.0)	0.3	0.1	0.9
*An. stephensi* (CE)	3.3 (2.7–3.7)	14.5 (13.4–15.8)	219.3 (179.9–295.0)	2.0 ± 0.1	71.4 (0.0)	2.6	0.0	1.0
*An. stephensi* (NCE)	11.0 (10.1–11.9)	46.1 (40.8–53.8)	618.3 (413.3–1037.8)	2.1 ± 0.1	5.7 (1.0)	0.2	0.0	1.0
*An. stephensi* (PE)	10.8 (9.2–12.3)	196.8 (126.4–382.1)	38,293.4 (10,121.7–287,360.5)	1.0 ± 0.2	4.7 (1.0)	0.2	0.0	1.0
*Cx. quinquefasciatus* (CE)	4.0 (3.2–4.5)	17.9 (16.1–20.1)	270.6 (220.0–401.7)	2.0 ± 0.1	100.1 (0.0)	3.6	0.0	0.9
*Cx. quinquefasciatus* (NCE)	6.1 (5.2–7.0	53.1 (45.2–64.9)	2672.8 (1471.3–5796.9)	1.4 ± 0.1	8.3 (1.0)	0.3	0.0	1.0
*Cx. quinquefasciatus* (PE)	10.2 (8.7–11.5)	126.6 (88.8–213.0)	12,340.4 (4138.7–62,329.9)	1.2 ± 0.1	1.7 (1.0)	0.1	0.0	1.0

ET_10_/ET_50_/ET_99_, Escape time 10/50/99%; 95% CI, 95% confidence interval; CE/NCE/PE, contact/non-contact/placebo exposure

**Fig. 3:**
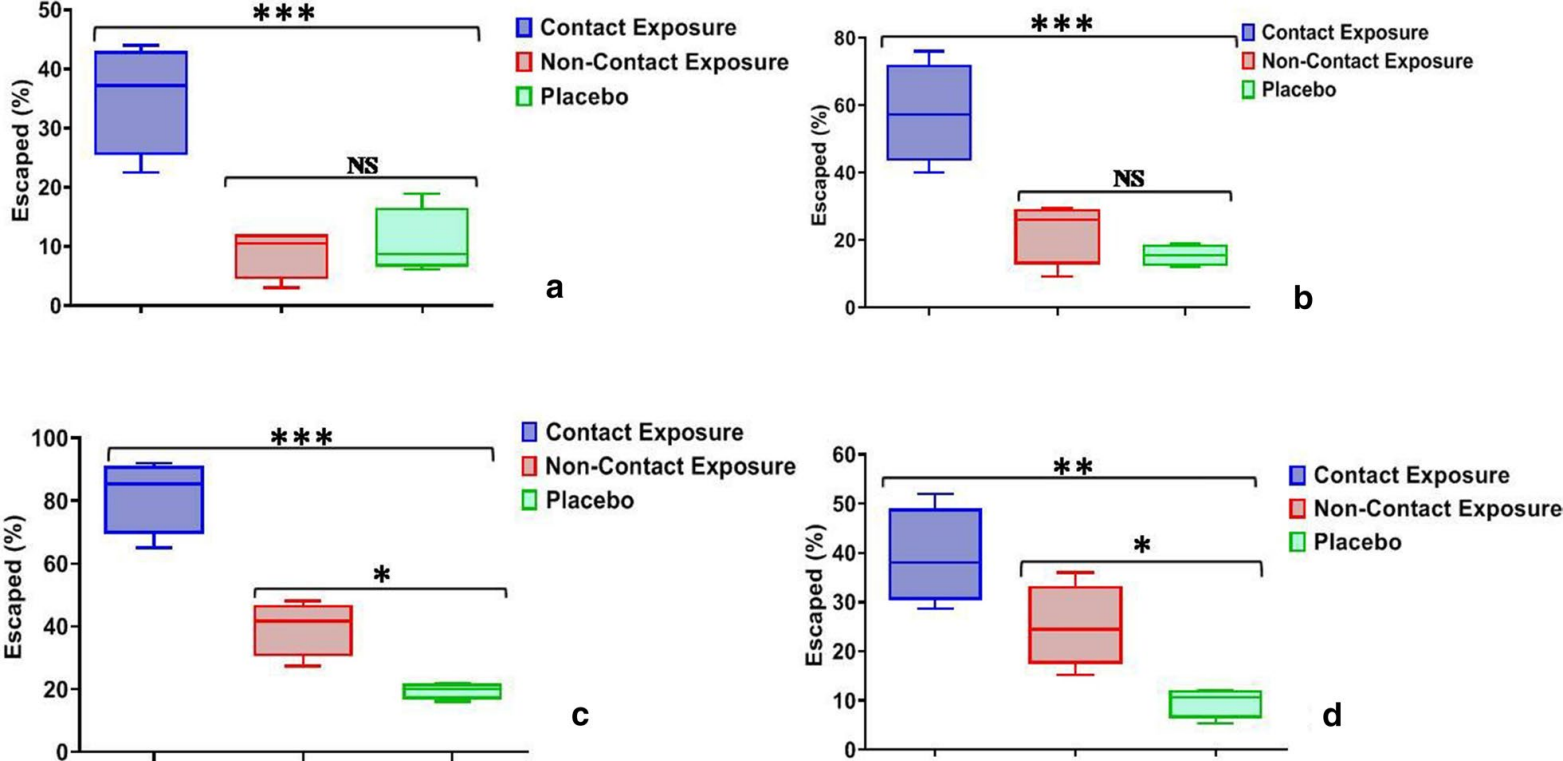
Box-whisker plot displaying the escape % of *Anopheles stephensi* mosquitoes **a** 10 min post exposure, **b** 20 min post exposure, **c** 30 min post exposure; **d** 24-h mortality (*p < 0.05; **p < 0.001; ***p < 0.0001; *NS* non-significant)

Malaria vector *An. stephensi* displayed an escape % of 82.0 ± 5.9 post 30 min of exposure compared to 35.2 ± 4.7 post 10 min of exposure in CE experiments. On the other hand, the escape % for NCE and PE post 30 min exposure was found to be 39.6 ± 4.4 and 19.5 ± 1.3 respectively (Table 1). The escape rate of *An. stephensi* at each time interval was statistically higher for CE compared to the other exposures (F ≥ 18.7; *p* ≤ 0.001, R^2^ ≥ 0.81) (Fig. 3). Furthermore, the escape rates in NCE and PE differ statistically post 30 min of exposure (t = 5.8; *p* = 0.01); however, after 10 min and 20 min exposure, these rate were found to be similar (t < 2.1; *p* > 0.12). Probit analysis showed that the ET_50_ of *An. stephensi* for CE was 14.5 min compared to 46.1 min in NCE and 196.8 min in PE respectively. The probit model further suggested that ET_10_, ET_50_ and ET_99_ calculation displayed deviation from linearity for CE (χ^2^ = 71.4; *p* = 0.0) but followed the linear pattern for NCE (χ^2^ = 5.7; *p* = 1.0) and PE (χ^2^ = 4.7; *p* = 1) (Table 2).

**Fig. 4:**
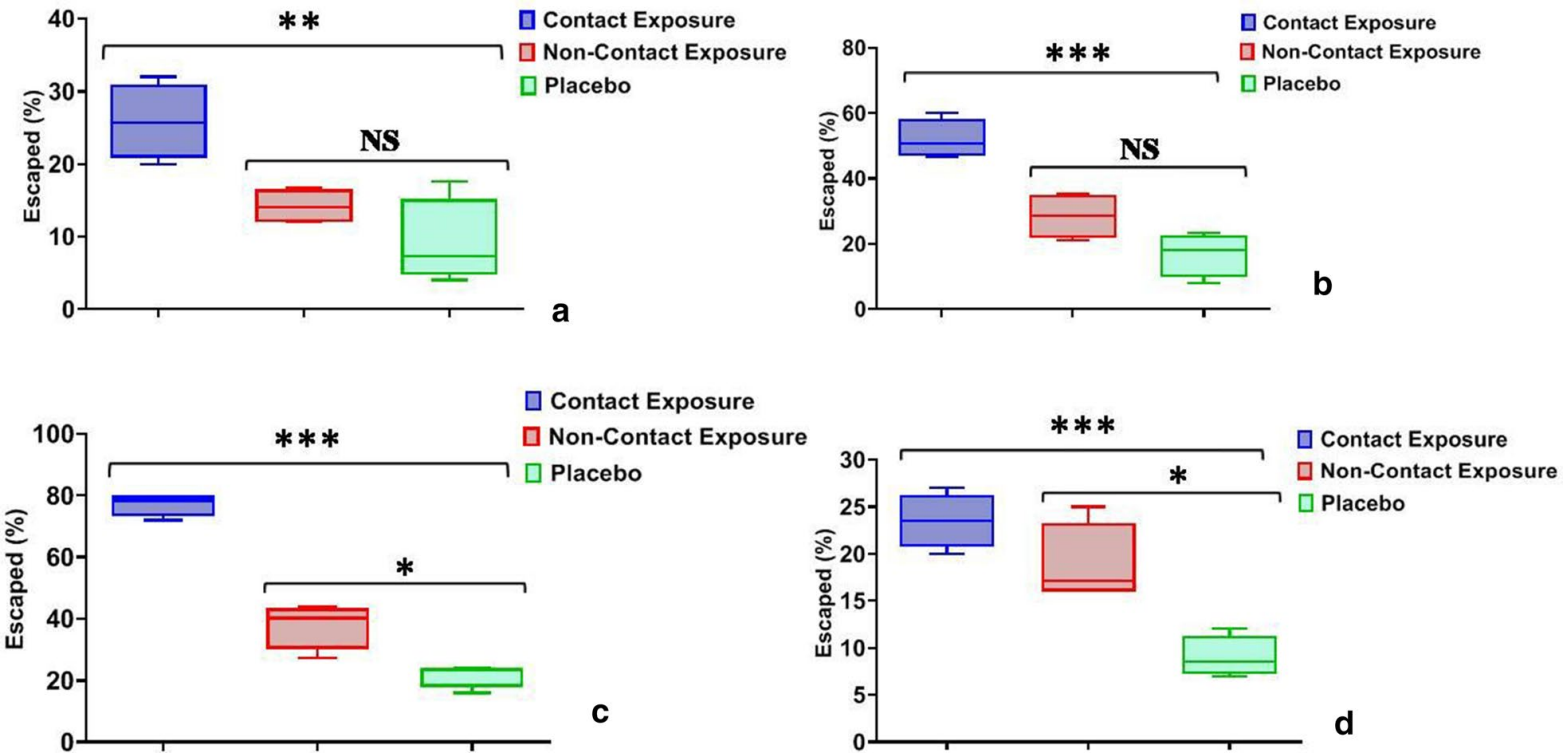
Box-whisker plot displaying the escape % of *Culex quinquefasciatus* mosquitoes: **a** 10 min post exposure, **b** 20 min post exposure, **c** 30 min post exposure; **d** 24 h mortality (*p < 0.05; **p < 0.001; ***p < 0.0001; *NS* non-significant)

The escape rate for *Cx. quinquefasciatus* mosquitoes post 10 min and 30 min of exposure was 25.8 ± 2.6 and 77.2 ± 1.9 respectively in CE (Table 1; Fig. 4) compared to 14.2 ± 1.3 and 37.9 ± 3.7, and 9.1 ± 2.9 and 21.7 ± 1.9 in NCE and PE respectively. A statistically higher number of *Cx. quinquefasciatus* mosquitoes escaped in CE than in the other exposure groups at 10 min (F = 12.8; *p* = 0.002; R^2^ = 0.74), 20 min (F = 29.7; *p* = 0.00; R^2^ = 0.87) and 30 min exposure (F = 116.6; *p* < 0.0001; R^2^ = 0.96) respectively. However no difference was observed in the escape rates in NCE and PE after 10 min and 20 min (*p* ≥ 0.1; t ≤ 2.4), while higher movement was recorded in NCE post 30 min of exposure (*p* = 0.03; t = 4.0) (Fig. 4). Log dose probit calculation showed (Table 2) that ET_50_ was 17.9 min for CE, 53.1 min for NCE and 126.6 min for PE respectively. Furthermore, the model suggested that escape time (ET_10_, ET_50_ and ET_99_) followed a linear pattern of escape for NCE (χ^2^ = 8.3; *p* = 1.0) and PE (χ^2^ = 1.7; *p* = 1.0) treatments, but did not follow this pattern in CE (χ^2^ = 100.1; *p* < 0.001).

Altogether in CE, the escape rates were stronger in *An. stephensi* mosquitoes at different time intervals compared to *Ae. aegypti* and *Cx. quinquefasciatus* mosquitoes but did not differ statistically (*p* ≥ 0.07). In NCE, *Cx. quinquefasciatus* tend to escape immediately as the escape rates were high after 10 min (F = 5.4; *p* = 0.03), bu did not differ after 30 min (*p* ≥ 0.09) compared to the other mosquito species. For PE, *Cx. quinquefasciatus* responded strongly after 30 min of exposure (F = 7.1; *p* = 0.01).

### Twenty-four-hour delayed mortality

Results suggested that in all the tested mosquito species, 24-h delayed mortality was higher in CE. For *Ae. aegypti*, although the mortality was higher in CE (27.4 ± 1.6; 95% CI: 20.4–34.4) (F = 71.1; *p* < 0.0001), but found to be similar to NCE (t = 3.3; *p* = 0.08), whereas in *An. stephensi* and *Cx. quinquefasciatus* mosquitoes, the delayed mortality was statistically higher in the CE compared to the other exposure groups (Table 1). Among the tested mosquito species, the 24-h delayed mortality was found to be statistically higher (F = 6.4; *p* = 0.02) in *An. stephensi* for CE, but did not vary for NCE (*p* ≥ 0.3) and PE (*p* = 0.6) treatments among the tested mosquito species.

### Survival analysis

**Fig. 5:**
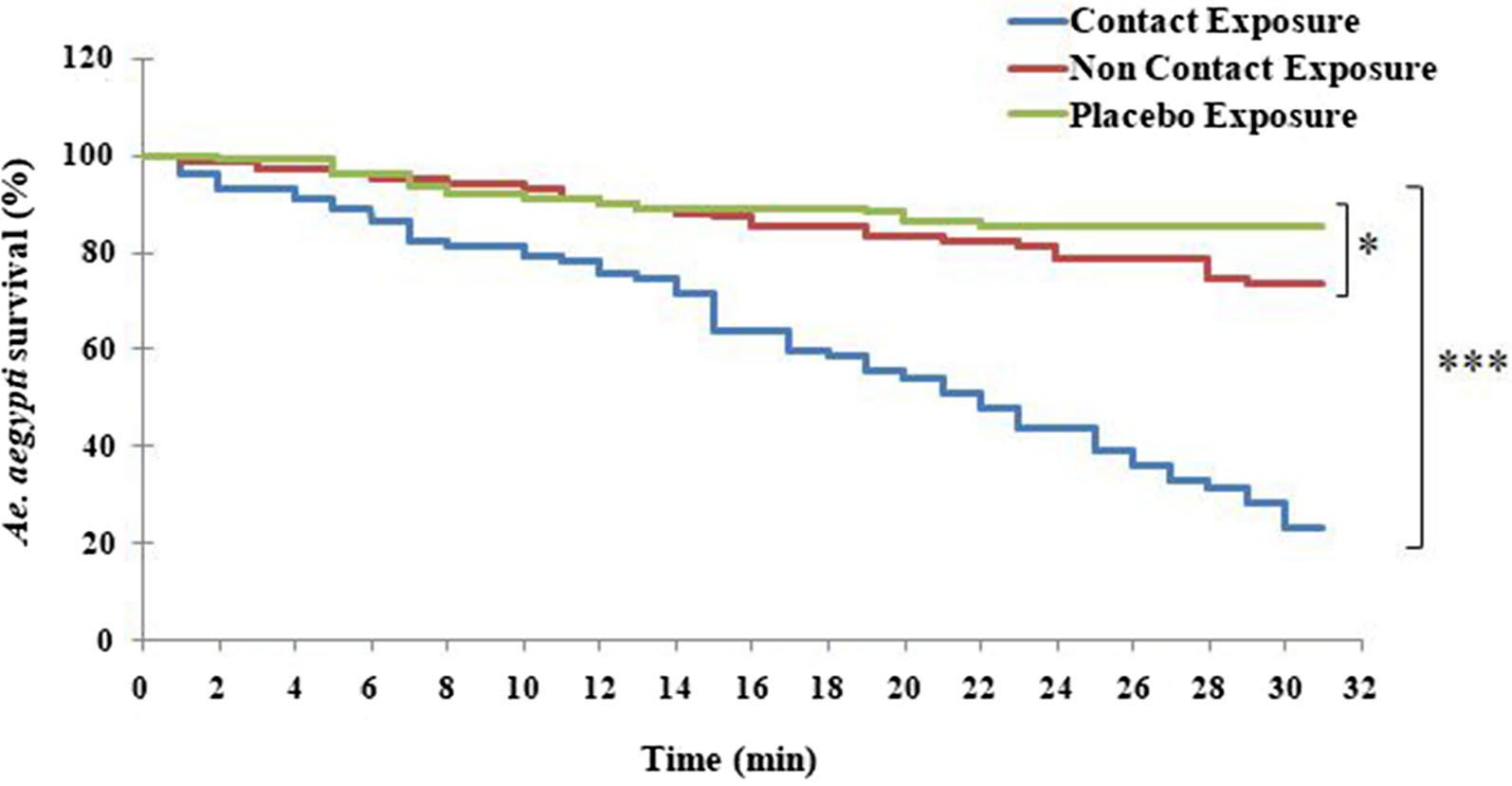
Survival analysis showing recurrence-free movement of *Aedes aegypti* mosquitoes in the escape chamber for CE, NCE and PE. The survival curve showed differences among the three exposure groups (*p < 0.05; ***p < 0.0001)

**Fig. 6:**
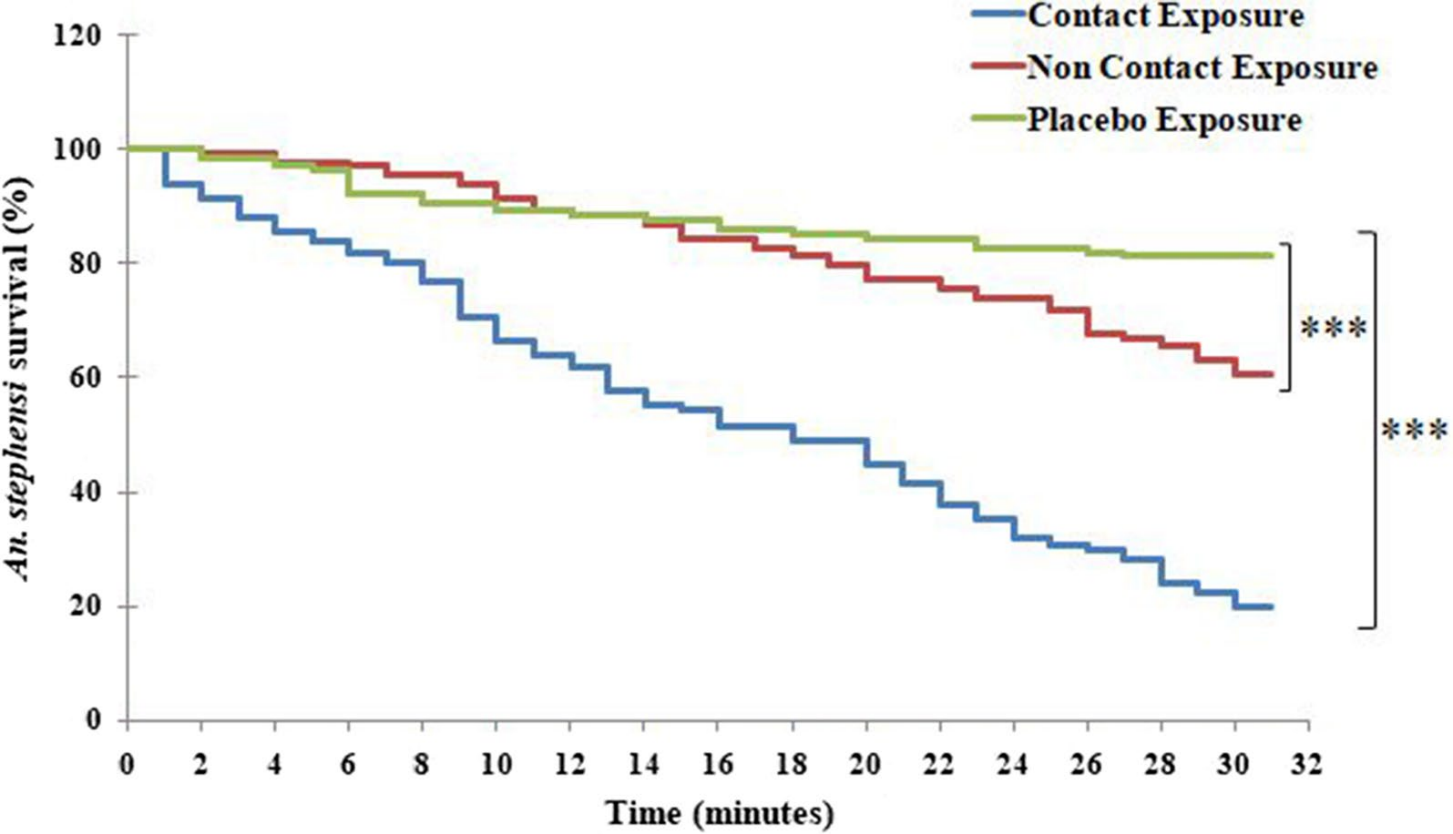
Survival analysis showing recurrence-free movement of *Anopheles stephensi* mosquitoes in the escape chamber for CE, NCE and PE. The survival curve showed differences among the three exposure groups (***p < 0.0001)

**Fig. 7:**
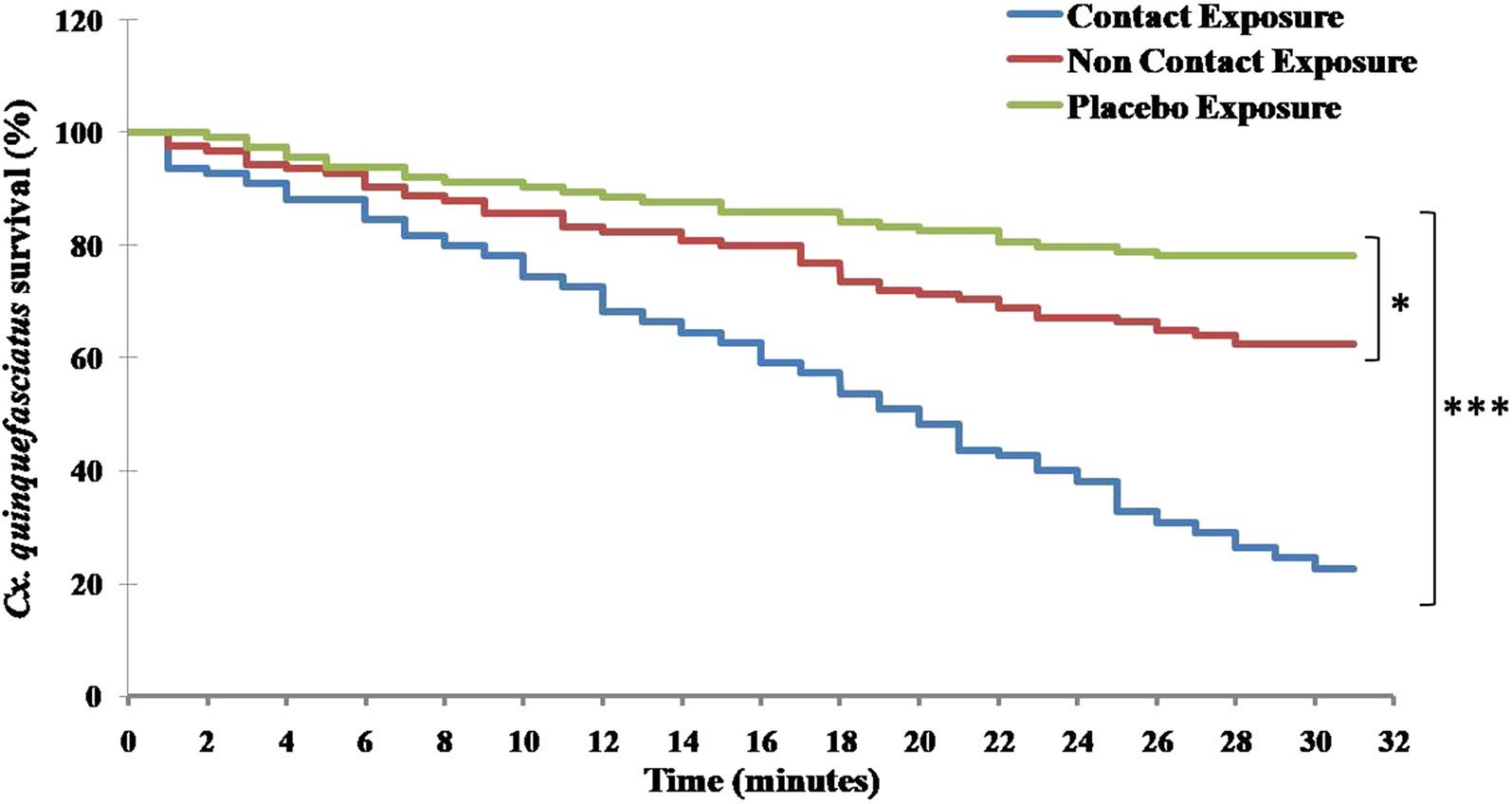
Survival analysis showing recurrence-free movement of *Culex quinquefasciatus* mosquitoes in the escape chamber for CE, NCE and PE. The survival curve showed differences among the three exposure groups (*p < 0.05; ***p < 0.0001)

Survival analysis showed that after 30 min of exposure, 23.5%, 73.8% and 85.7% of *Ae. aegypti* remained in the exposure chamber for CE, NCE and PE respectively (χ^2^ = 96.7; *p* < 0.0001). The patterns of escape response for tested species in different exposure groups using survival probability during 30 min exposure are shown in Figs. 5, 6 and 7. For NCE and PE, survival (those remaining in the exposure chamber) was higher in PE compared to NCE post 30 min of exposure (χ^2^ = 4.1; *p* = 0.04) (Fig. 5). Similarly, different escape responses were recorded for CE, NCE and PE in *An. stephensi*. It was found that a statistically lower number of *An. stephensi* mosquitoes survived in CE compared to other exposures (χ^2^ = 98.0; *p* < 0.0001). Also, the survival rates were significantly lower in NCE than in PE (χ^2^ = 11.9; *p* < 0.001) (Fig. 6). A similar response was also obtained for *Cx. quinquefasciatus*. It was observed that the survival rates were higher in PE compared to the other exposures (χ^2^ = 73.6; *p* < 0.0001) (Fig. 7). Furthermore, the survival response also differed between NCE and PE (χ^2^ = 6.2; *p* = 0.01).

### HPLC quantification of active ingredients

**Fig. 8:**
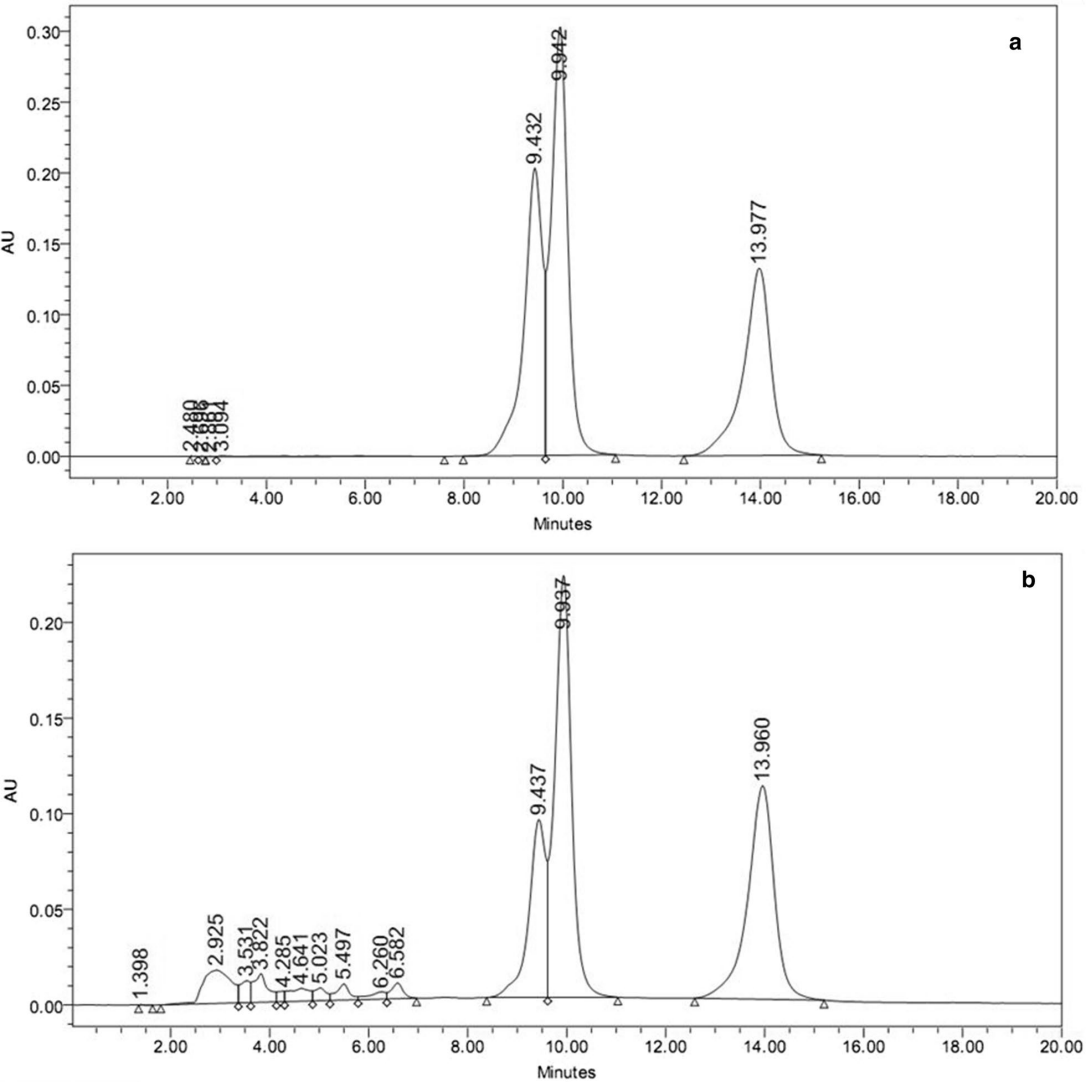
HPLC estimation of active components in FRSRIP: **a** HPLC-UV chromatogram of standards; pyriproxifen (9.432 min), chlorpyriphos (9.942 min) and deltamethrin (13.977 min); **b** HPLC-UV chromatogram of the paint sample; pyriproxifen (9.437 min), chlorpyriphos (9.937 min) and deltamethrin (13.960 min)

The HPLC method developed could separate and estimate all three active ingredients simultaneously in duplicate. The HPLC chromatograms of the standards and paint sample are represented in Fig. 8. Estimation of all three active ingredients performed through respective calibration curves showed that the contents (%w/w) of deltamethrin, chlorpyriphos and pyriproxyfen were 1.129 ± 0.025, 0.542 ± 0.038 and 0.114 ± 0.003 respectively. The estimated concentrations were higher than the added values. This may be attributed to drying of paint during which the volatile mineral turpentine oil (MTO) was evaporated leaving behind the non-volatile components of the paint. Hence, the effective concentration of the active ingredients increased in the dried paint.

## Discussion

A slow-release insecticidal paint formulation has been developed with the primary objective of killing of mosquitoes that come in contact with the treated surfaces. This formulation has been developed primarily for use by forces operating in endemic areas with high mosquito and other arthropod prevalences. This activity of the formulation has been evaluated and found effective up to 2 years after application (Dhiman et al. unpublished data). This formulation has also been evaluated in military locations and found effective in containing different arthropods. However, in part inspired by the excito-repellency activity of the active component deltamethrin used in the formulation, the study was designed to assess the contact and non-contact responses of different mosquito vectors [8].

The results suggested that there is an unambiguous avoidance response in all the three tested vector mosquito species in the contact exposures. The excito-repellency response was elicited with the start of exposure and within 10 min of exposure > 20% of mosquitoes had escaped. Mosquito vectors displayed striking excito-repellency with the passage of time, and after 30 min > 75% repellency could be observed. Relatively high escape response was observed in *An. stephensi* during the initial phase of CE compared to *Ae. aegypti* and *Cx. quinquefasciatus.* However after 30 min of CE the response obtained was similar among the three species. On the other hand, in NCE, *Cx. quinquefasciatus* initially showed high escape reactions compared to the other two species, while the response of *Ae. aegypti* was found to be reduced and delayed. Furthermore, reduced mortality was observed in *Cx. quinquefasciatus* after 24 h of exposure compared to the other tested species in both contact and non-contact experiments.

The relative difference among the mosquito species to respond to the insecticides in a given time with or without physical contact could be an inherent ability developed through a long evolutionary process. Therefore, the degree of excito-repellency response elicited by mosquitoes may differ depending upon the exposed species and the insecticides used in the formulation. Pyrethroids may exhibit multiple behavioural effects on mosquitoes at the same time. These could prevent mosquito entry into a structure, elicit excito-repellency and irritancy, and produce delayed as well [25]. Studies have attempted to demonstrate the response elicited by intradomicilary insecticide use and showed that considerable avoidance is observed, although it may vary among different species exhibiting different levels of insecticide susceptibility [15, 26–28]. A declining escape response can be found when mosquitoes are knocked down immediately in the exposure chamber because of the stronger knockdown effect at higher concentrations of insecticide [19]. However, in the present study the mosquitoes did not display any significant knockdown during the exposure period but recorded considerable delayed mortality after 24 h. The repellency responses observed in the present study were similar to those recorded previously where > 75% repellency was observed in *An. minimus* mosquitoes [15]. An earlier field study conducted in the malaria-endemic north-east border region of India [27] compared malaria vector density of mosquitoes collected from insecticide-unsprayed temporary structures and sprayed areas such as walls, roofs, pillars, etc., to demonstrate the behavioural resistance. The study revealed that the malaria vector *An. annularis* avoided landing on insecticide-treated surfaces.

The presently developed SRIP formulation contains deltamethrin and chlorpyriphos as active insecticide molecules and demonstrated strong excito-repellent activity against some of the known laboratory-colonised mosquito vectors species. Some previous studies have shown that long-colonised populations of mosquito vectors have a lower avoidance behaviour response to synthetic pyrethroid exposure compared to the recent wild-caught populations [28, 29]. However these studies did not take account the insecticide susceptibility status of the tested populations [12].

The tested mosquito species displayed considerable delayed mortality post 24 h of observation in CE, suggesting that they probably came in contact with the formulation for a brief time and then escaped to the escape chamber. On the other hand, in case of NCE, the mosquitoes received insecticide cues but did not get the chance to have direct contact; therefore, comparatively less delayed mortality could be observed. This suggests that in addition to excito-repellency, delayed mortality has a considerable effect when mosquitoes have the choice to contact the formulation. The present study observed high mortality compared to an earlier study [15] from Thailand which used insecticide-treated papers to demonstrate the excito-repellency and reported maximum mortality of 13.3% (0–13.3%) for escaped *Anopheles* mosquitoes but 100% (43–100%) for non-escaped mosquitoes. It has been argued that the mortality effect of insecticide-based formulations ceases with time because of a decline in the concentration of the active ingredients [26]. However, the excito-repellency deterrence may not decline with time as it is not a function of a high dose of the active ingredient. Therefore, excito-repellency-induced avoidance is likely to have epidemiological significance because of the reduced risk of overall mosquito bites to humans.

Survival analysis suggested that > 75% of tested mosquitoes displayed physiological avoidance in CE. However, the survival response relationship between the mosquito percent remaining in the treatment and those escaped at a 1-min interval was found similar among all three tested species. These findings were similar to an earlier study which demonstrated that the escape response in dengue vector *Ae. aegypti* against repellents was similar for contact and non-contact exposures [12]. It has been shown that mosquito vectors that come in direct contact even for a brief period of time or those which come in contact with insecticide molecules present in space in the insecticide formulation-applied areas become excited and display locomotive behaviour, often resulting in premature movement away from the insecticide-treated areas [14]. Therefore, limiting insecticidal action to knockdown or killing is not adequate, but it is equally pertinent to describe the excito-repellency of such a formulation to understand the overall impact on target vectors. It is obvious that the initial concentration of insecticides used in the formulations aimed at achieving an insecticidal effect decline with time. However, at sub-lethal concentrations these insecticides are able to induce irritant responses in vector mosquitoes and enforce avoidance from treated surfaces [9–11].

Most of interventions used in vector control operations employ single insecticide-based formulations; however, a formulation using an optimised mixture of insecticides belonging to different groups could be most useful in resistance management in endemic areas. Furthermore, new approaches to delivering insecticides to control vector mosquitoes compared to conventional approaches are needed at present. The paint formulation may deter the indoor resting mosquitoes and provide protection when the users are engaged in other household activities and not using intervention measures. Therefore, the currently tested paint formulation may lead to overall improvement of human living areas in addition to having sustained efficacy for a longer time.

## Conclusion

Although, there was variation in the excito-repellency response and delayed mortality among the mosquito species, altogether, the study findings demonstrate the strong excito-repellency response elicited by three major mosquito vectors. The combination of insecticides used in the formulation will have the added advantage that the lethal effect of chlorpyriphos and deltamethrin coupled with the strong excito-repellency response of deltamethrin will broaden the overall impact spectrum in protecting users from mosquito bites. The excito-repellency data generated in the present study provide crucial information on the effectiveness of the tested formulation and could be useful in the reducing transmission intensity and disease risk in endemic countries.

## Data Availability

The formulation intellectual property right (IPR) has been filed. All relevant data on the study are available from the corresponding author on reasonable request.
